# Subpubic cartilaginous cyst: a rare cause of vulvar lesion

**DOI:** 10.1590/0100-3984.2017.0185

**Published:** 2019

**Authors:** Claudio Marcio Amaral de Oliveira Lima, Antônio Carlos Coutinho, Roberta Araújo de Arruda Câmara

**Affiliations:** 1 Clínica de Diagnóstico Por Imagem (CDPI) e Clínica de Diagnóstico por Imagem Fátima Digittal, Rio de Janeiro, RJ, Brazil.

*Dear Editor,*

**Figure 1:**
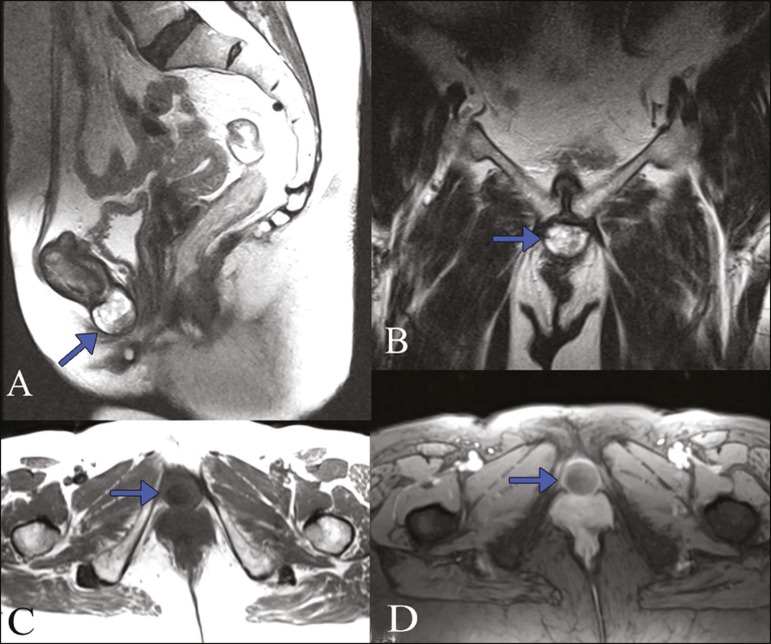
MRI of the pelvis with a special focus on the pubic area. Sagittal and coronal T2-weighted sequences (**A** and **B**, respectively), axial T1-weighted sequence (**C**), and T1-weighted sequence with fat-suppression after gadolinium administration (**D**). Formation with a cystic aspect and a heterogeneous signal that was predominantly hyperintense in T2-weighted sequences and hypointense in T1-weighted sequences, the formation showing thickened walls and contrast enhancement (arrows).

A 67-year-old postmenopausal, multiparous female presented with a one-month history of vulvar edema, with no vaginal bleeding or dysuria, reporting only local discomfort. A solid, fixed, painless nodule was found on her vulva. Magnetic resonance imaging (MRI) showed an oval fibrous mass, with a hypointense signal in T1-weighted sequences and a heterogeneous, predominantly hyperintense signal in T2-weighted sequences (Figure 1). The formation showed thick walls and contrast enhancement, with no restricted diffusion. It was in close contact with the lower edge of the symphysis pubis and measured 2.8 × 2.5 × 2.3 cm. Based on the MRI findings and the location of the lesion, we considered a diagnosis of subpubic cartilaginous cyst (SCC).

The first description of SCCs was in 1996 by Algucial-Garcia et al.^(^^1^^)^. In the international literature, only 12 cases of SCC have been reported^(^^2^^)^. Almost all of those cases involved multiparous women, between 50 and 80 years of age, with a vulvar mass, although with various presentations^(^^2^^)^: as a painful mass, in four cases; as a painful mass accompanied by abdominal pain, in three cases; as urinary dysfunction, in another four cases; and as pain at the base of the penis with sexual dysfunction, in a rare case involving a male patient.

An SCC is a rare form of ganglion cyst that begins on the inferior surface of the symphysis pubis, consisting of a collagen capsule composed of gelatinous fibrocartilaginous tissue in degeneration, mucin, and debris^(^^2^^-^^5^^)^. It is believed to be secondary to degenerative changes. It may remain stable or present minimal size reduction with only one case in 2015 in Japan, where there was complete and spontaneous regression after two years^(^^2^^-^^5^^)^.

Imaging exams, MRI in particular, have garnered increasing attention in the assessment of pelvic diseases^(^^6^^-^^10^^)^. A diagnosis of SCC, which is based on clinical and imaging findings, depends on the amount of mucinous and cartilaginous material, which results in a heterogeneous aspect on MRI^(^^2^^,^^11^^)^. Degenerative alterations in the symphysis pubis can be seen on X-rays^(^^3^^)^.

The MRI findings of SCC were first described in 2004 by Kim et al.^(^^12^^)^: Such findings include a signal that is hypointense (in relation to that of the muscle) in T1-weighted sequences and heterogeneously hyperintense in T2-weighted sequences, the lesion being in close, extensive contact with the symphysis pubis and presenting wall enhancement after gadolinium administration, without internal enhancement^(^^2^^)^.

The clues for a correct diagnosis of an SCC are a cystic lesion that is located on the midline and is in close contact with the symphysis pubis^(^^5^^,^^11^^)^. The differential diagnoses of SCC in patients with a vulvar mass include lipomas; cysts of the urethra; Nabothian cysts; Bartholin gland cysts; Gartner duct cysts; paratubal cysts; cysts in the symphysis; subchondral pseudocysts in rheumatoid arthritis; and subchondral cysts. Other potential diagnoses include malignant tumors such as squamous cell carcinoma, Bartholin gland carcinoma, chondrosarcoma, and melanoma of the vulva^(^^2^^,^^5^^,^^12^^,^^13^^)^. In general, the differentiation is easy, depending, as it does, on the location and radiological characteristics of the lesion^(^^2^^,^^12^^,^^14^^)^.

Preoperative biopsies of SCCs are reserved for cases in which there is a high suspicion of malignancy^(^^1^^,^^6^^)^. The treatment of choice is resection, because the bulky content of the cyst precludes aspiration. No cases of recurrence have been reported, although none of the patients involved were followed for more than three years. In one case, the SCC was not treated and there were no changes in its size or characteristics after two years of follow-up. In that case, the resection of the SCC was complicated by separation of the symphysis pubis^(^^1^^,^^11^^)^. Because SCC is a benign condition, all efforts should be made to preserve the stability of the symphysis pubis^(^^2^^,^^4^^,^^14^^)^.
